# Long-term Local Control of Primary Non-small Cell Lung Cancer and Adrenal Oligometastasis with Stereotactic Body Radiotherapy: A Case Report

**DOI:** 10.7759/cureus.4188

**Published:** 2019-03-06

**Authors:** Victoria Krzywicki, Darin D Gopaul

**Affiliations:** 1 Miscellaneous, Poznań University of Medical Sciences, Poznań, POL; 2 Oncology, Grand River Hospital, Kitchener, CAN

**Keywords:** oligo-metastatic, non-small cell lung cancer, stereotactic body radiotherapy (sbrt), adrenal metastasis, lung cancer

## Abstract

Treatment options for non-small cell lung cancer (NSCLC) patients presenting with synchronous adrenal oligometastases (stage IV disease) include local treatment such as surgery, stereotactic body radiotherapy (SBRT) or systemic treatment such as chemotherapy. A case of successful SBRT treatment to a primary NSCLC with a synchronous left adrenal oligometastasis achieving long-term local control of both lesions is reported.

## Introduction

Patients with non-small cell lung cancer (NSCLC) frequently present with metastatic disease (stage IV), which may be treated with palliative chemotherapy or palliative radiotherapy. A subset of patients that present with primary NSCLC and three to five extrathoracic metastatic lesions are considered to be in an oligometastatic state [1]. In such situations, local curative treatment may result in long-term disease-free survival.

Distant metastases to the adrenal gland from a primary NSCLC is common. Standard treatments for medically operable patients include surgical resection of primary tumours and subsequent adrenalectomies. These have produced high local control rates and increased overall survival (OS) [2]. Stereotactic body radiotherapy (SBRT) is an increasingly available, non-invasive option.

A case of a patient with oligometastatic stage IV NSCLC treated only with SBRT to both the primary and one adrenal metastasis is discussed. Informed consent for the publication of this case report was obtained from the patient.

## Case presentation

**Figure 1:**
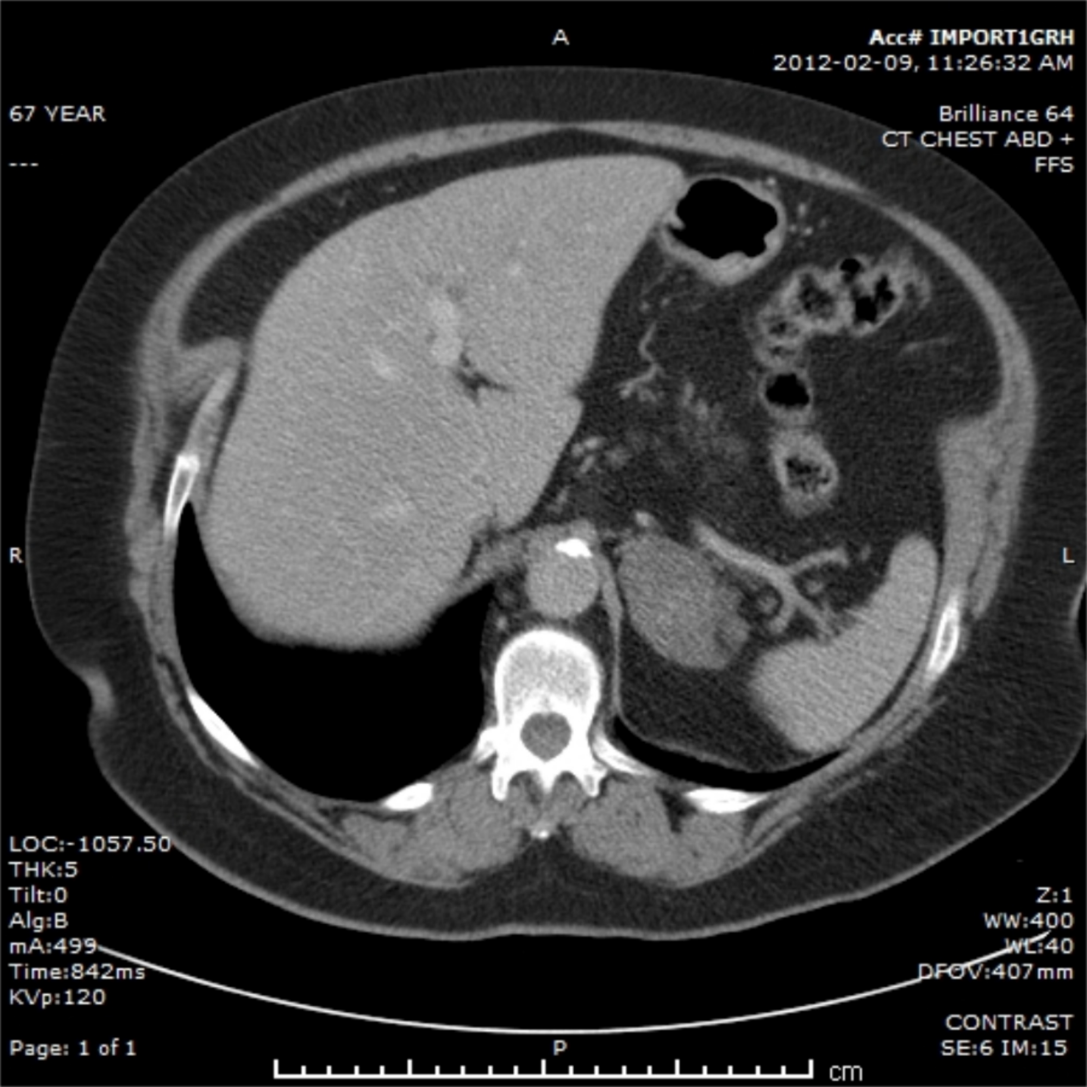
Initial computed tomography (CT) scan of the adrenal oligometastasis.

A 67-year-old female sought medical attention for an initially suspected diagnosis of pneumonia in January 2012. A chest X-ray was performed and revealed a suspicious mass in the right upper lobe of the lung. A computed tomography (CT) scan of the abdomen and chest demonstrated a 3.1-cm lesion in the right upper lobe with no hilar or mediastinal lymphadenopathy and a suspicious left adrenal mass, measuring 5.2 cm (Figure 1).

The patient underwent a biopsy for the lung lesion which confirmed a poorly differentiated adenocarcinoma which was also positive for thyroid transcription factor 1 (TTF-1). A staging whole body 18F-fluorodeoxyglucose (FDG) positron emission tomography (PET) scan showed uptake in the right lung mass (SUV 29), and the left adrenal gland mass only (8.9). The patient was not considered a surgical candidate, which led to a referral for radiation therapy.

The patient presented to the clinic in May 2012 with complaints of mild shortness of breath upon exertion. No complaints of cough, hemoptysis, chest pain, anorexia or weight loss were reported. The patient had a history of high blood pressure and is a 40-pack year smoker. Pulmonary function testing demonstrated decreased vital capacity 1.6 L (62% predicted), forced expiratory volume in one second (FEV1) 1.0 L (49% predicted), and FEV1/forced vital capacity (FVC) ratio indicating obstructive disease. Diffusion lung capacity of carbon monoxide (DLCO) was within normal limits. Physical examination of the patient was unremarkable. The definitive diagnosis was primary adenocarcinoma of the right lung with an oligometastatic lesion to the left adrenal gland (stage IV).

**Figure 2:**
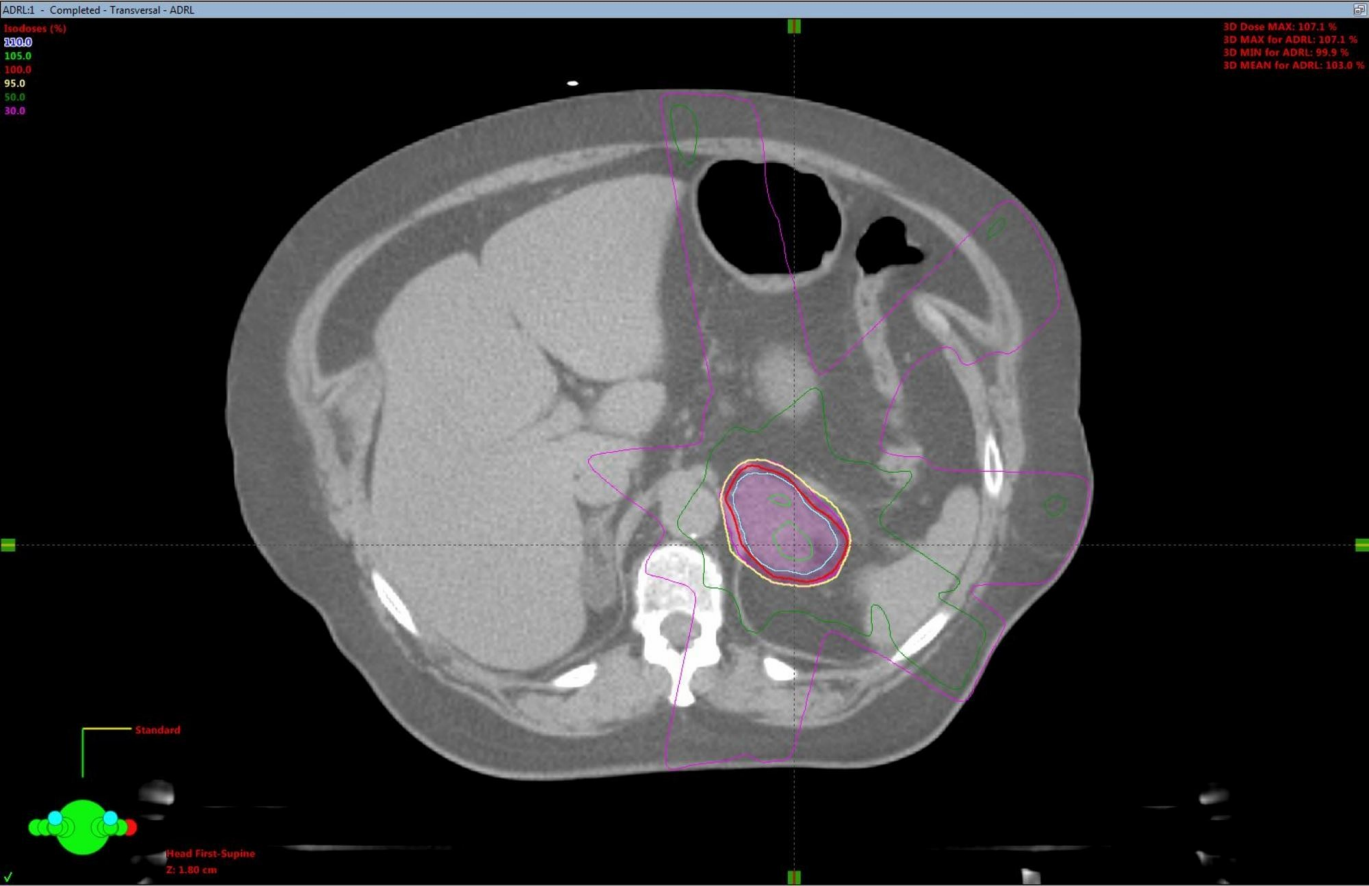
Adrenal oligometastasis stereotactic body radiotherapy (SBRT) planning computed tomography (CT) axial view.

**Figure 3:**
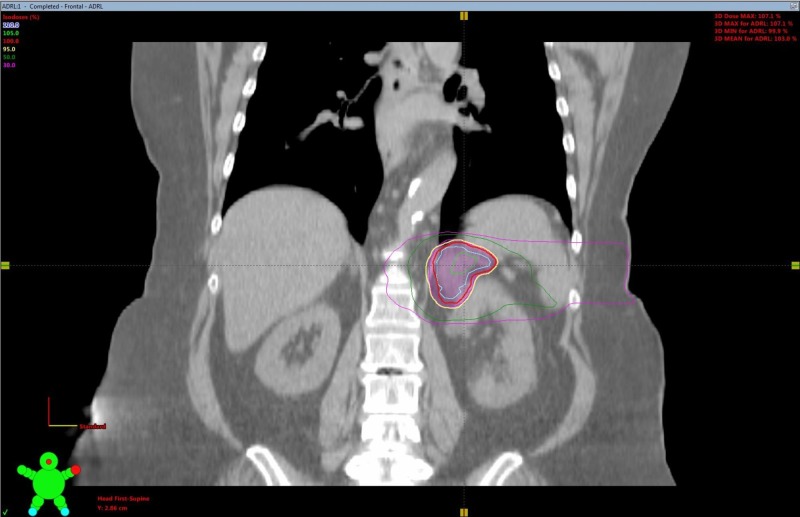
Adrenal oligometastasis stereotactic body radiotherapy (SBRT) planning computed tomography (CT) coronal view.

**Figure 4:**
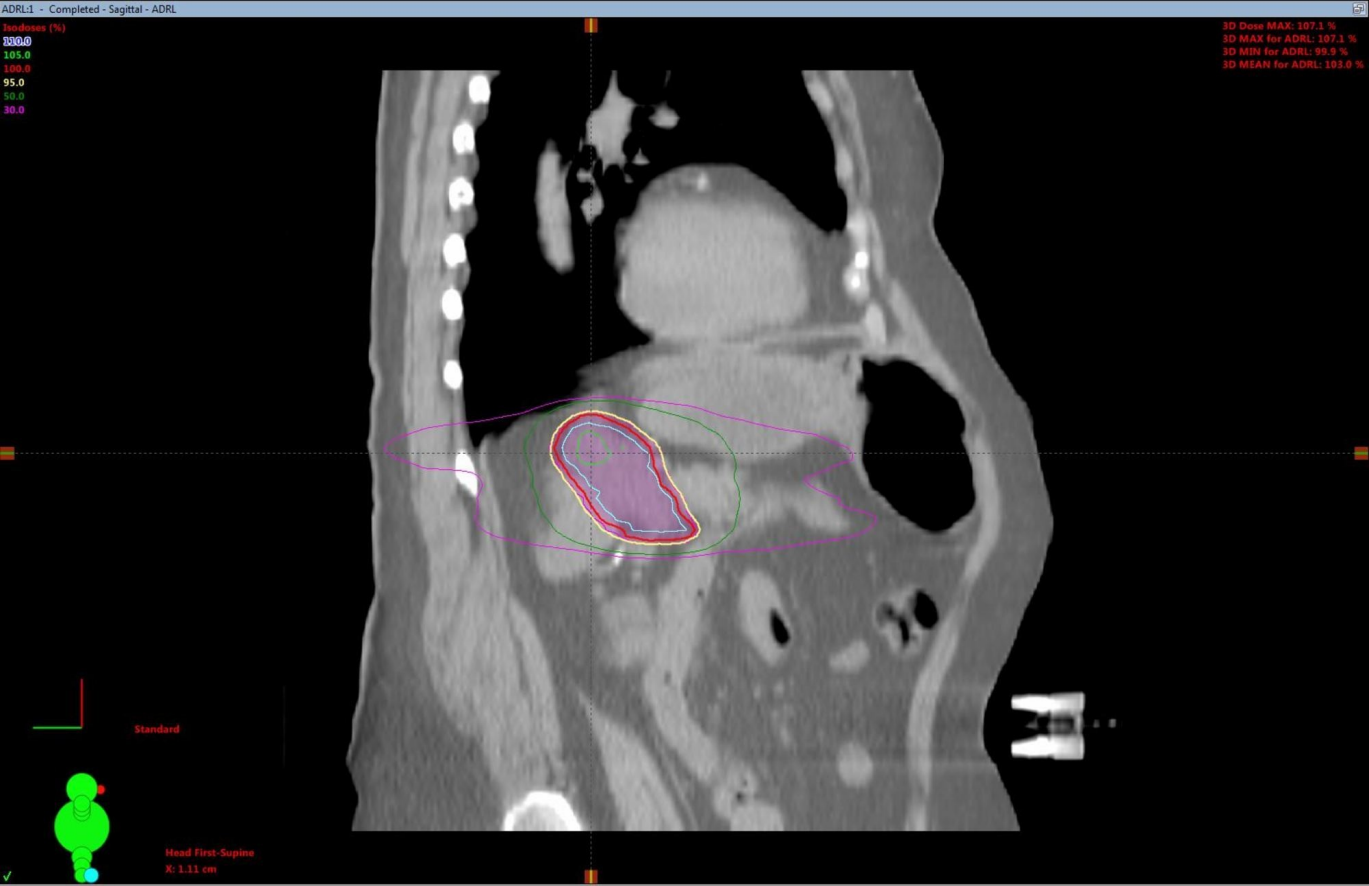
Adrenal oligometastasis stereotactic body radiotherapy (SBRT) planning computed tomography (CT) sagittal view.

**Figure 5:**
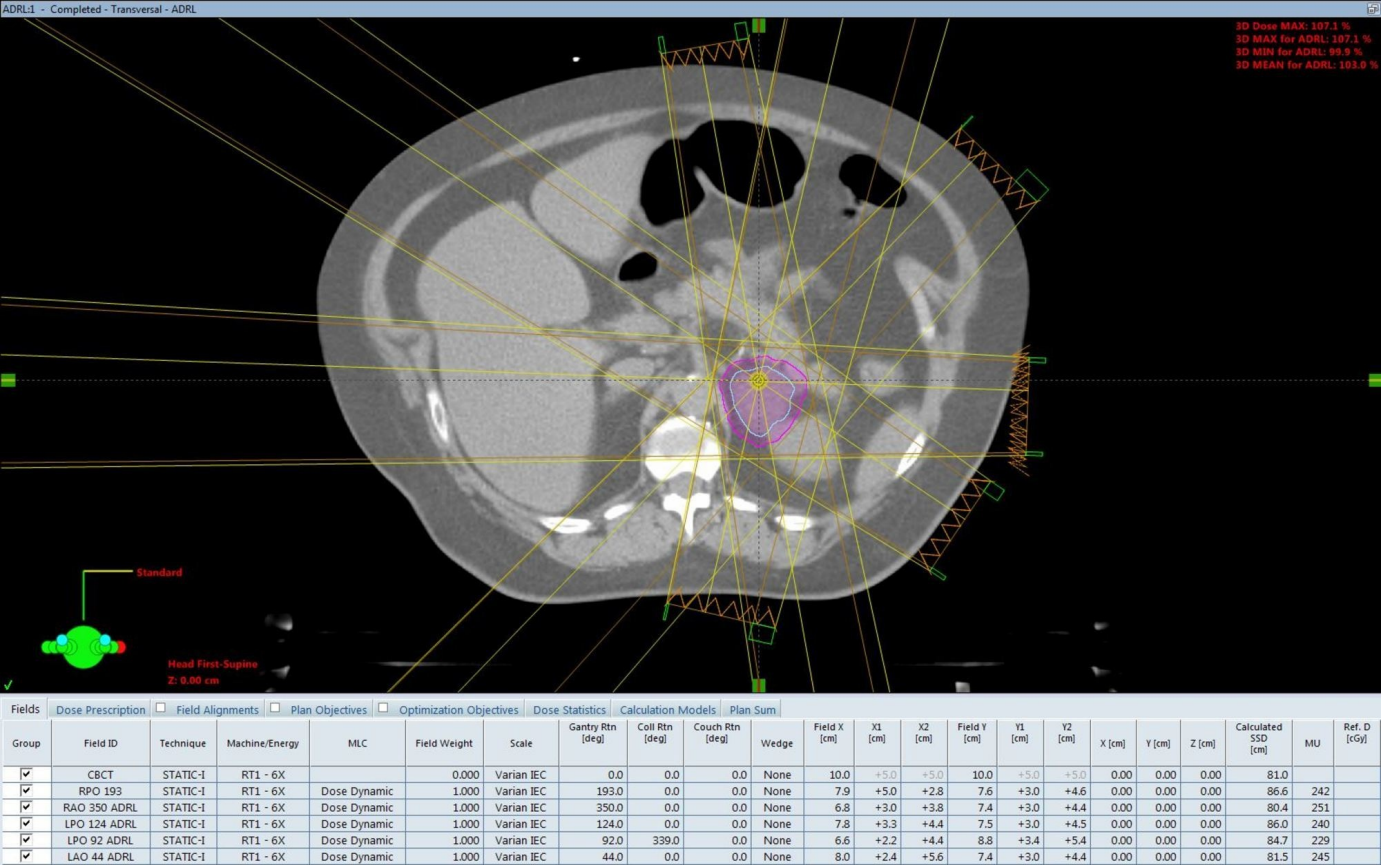
Adrenal oligometastasis stereotactic body radiotherapy (SBRT) planning computed tomography (CT) field arrangement.

SBRT treatment planning CT scans can be seen in Figures 2-5. The left adrenal mass was treated first in June 2012 followed by the right lung mass one month later. 4D CT simulation with abdominal compression was performed for each site. Cone-beam CT image guidance was used prior to each fraction.

A dose of 30 Gy over six fractions was delivered via linear accelerator to the left adrenal mass utilizing a five-field intensity-modulated radiation therapy (IMRT) technique with six MV photons, to a prescribed isodose of 100%. The right lung mass was treated with a nine-field non-coplanar SBRT technique with a dose of 48 Gy in four fractions over two weeks, prescribed to the 80% isodoses. Treatment concluded in August 2012. Treatment was well tolerated, with a short bout of nausea reported.

The patient was diagnosed with a high-grade superficial bladder cancer in September 2012. This was treated with intravesical bacillus Calmette-Guérin (BCG).

Over the next few years, the patient underwent biannual follow-up CT scans. A CT scan in March 2013, at eight months post radiotherapy, indicated both the right lung mass and the left adrenal mass had decreased, measuring 1.5 cm and 5.0 cm, respectively. Follow-up PET scan at that time demonstrated a metabolic response at both sites with no significant uptake at both sites, thus no evidence of metastatic disease. She remains free of recurrent lung cancer just over five years later.

During a routine follow-up CT scan in March 2016, an asymptomatic transverse fracture of the lateral aspect of the right third rib with areas of sclerosis and lucency involving the right second and fourth ribs was diagnosed. The patient did not present any clinical symptoms of the fracture, as it was only evident on CT scan. The patient did not report any recent trauma or hospitalizations that would suggest another cause of the rib fracture. A bone scan that was performed to rule out possible metastasis was negative. This confirmed the rib fracture was a radiation-induced rib fracture, a late complication from SBRT.

Most recent chest-abdomen-pelvic CT in April 2017 and follow-up chest X-ray in October 2017 demonstrated no pleural effusion, no focal consolidation or new lung masses to indicate local failure. The patient remains clinically stable with local control of the primary tumour and continues with routine follow-ups.

## Discussion

The development of distant metastasis in cancer progression is hypothesized by a few theories. The Halsted theory examined the spread of breast cancer and proposes a contiguous spread of cancer cells from the primary tumour to the first lymphatic node with subsequent orderly spread to the next regional lymph node in the chain and ending in distant sites [1]. The Systemic theory suggests that clinically evident cancer indicates widespread systemic involvement upon detection. Small tumours indicate early manifestations of systemic disease but can also be multiple or widespread (micrometastases) while lymph nodes act only as markers of distant disease and do not follow a pattern of step-wise involvement [1].

A third paradigm, the existence of an oligometastatic clinical state, was hypothesized by Hellman and Weichselbaum in 1996 which “argues that cancer comprises a biologic spectrum extending from a disease that remains localized to one that is systemic when first detectable but with many intermediate states” [1].

Today, an oligometastatic state in practice suggests an indolent tumour evolution with limited metastatic capacity exhibited by a limited number of metastases. Hellman and Weichselbaum also proposed, “patients with oligometastases either de novo or following systemic treatment, should be cured by ablation of these lesions” [1].

With this new classification of disease and understanding of tumour progression, locally ablative treatments became the new standard in these patients to achieve long-term disease-free survival. Since adrenal glands are a common site of distant metastasis in NSCLC, many studies stemming from this original hypothesis have concluded that curative treatment of the primary and metastatic lesion sites either by surgical resection or radiotherapy may increase overall survival [3].

The surgical resection of primary lung tumours or adrenal metastases (adrenalectomies) already has a highly established role in stage IV disease. The characteristic features of a surgical candidate include an M1b classification of adrenal oligometastasis, adequate performance status (especially pulmonary function in NSCLC patients), and minimal comorbidities. Barone et al. reported overall survivals in adrenal oligometastatic NSCLC patients who underwent adrenalectomies at 31 months, compared to those who underwent alternative therapies at 13 months [2].

Efforts to find reliable, and effective treatments for medically inoperable patients have increased, specifically concerning SBRT. This highly precise form of radiotherapy delivered in a hypofractionated form, has been adopted into modern clinical practice as a feasible alternative treatment option to increase symptom-free duration and long-term survival with a minimal side effect profile. In a study of NSCLC patients with isolated adrenal metastases treated by SBRT, the overall survival rate was reported at 23 months, similar to that of an adrenalectomy [4].

Clinically, adrenal metastases manifest as synchronous (discovery at time of diagnosis of primary tumour) or metachronous (discovery after a period of time from initial diagnosis). CT, PET, and magnetic resonance imaging (MRI) scans are the preferred method available to detect the metastases as 90%+ of patients are asymptomatic. The advancement of imaging techniques in recent years has improved the rate of correctly diagnosed adrenal oligometastases and thus has led to earlier intervention and increased survival rate. One study of 464 patients with metastatic disease in the adrenal glands, 149 of which had lung primaries, reported 67% of patients presenting with synchronous adrenal metastases, while the remaining patients’ metastases were detected over a median duration of seven months from primary tumour diagnosis. Only 4.3% (n = 20) of the total number of patients clinically presented with symptoms related to adrenal metastases before death, nine of which were patients with primary lung tumours [5].

There is limited literature available addressing the treatment of primary NSCLC patients presenting with synchronous adrenal oligometastases where both primary and oligometastatic lesions were treated only with radiotherapy. The success rate of SBRT in NSCLC primary lung tumours is often examined separately to that of outcomes in adrenal metastases treatment that may occur. The case presented proves the efficacy of SBRT as well as supports the proposed hypothesis that using aggressive, locally ablative treatments for oligometastatic NSCLC can result in effective local and long-term control with a minimal side effect profile.

Although cases of long-term survival (more than five years) in NSCLC adrenal oligometastatic patients have been reported, these have involved combinations of surgical resection of the primary tumour coupled with radiotherapy to the metastatic lesions [6]. The fascinating aspect of the case discussed is the survival of the patient at the time of writing for over five years treated solely with SBRT to the adrenal oligometastasis and the primary lung tumour, without systemic therapy. The future of SBRT is very promising as the amount of clinical evidence continues to grow, proving its success as one of the safest, non-invasive treatments available.

## Conclusions

SBRT to both the primary and oligometastatic lesions, if feasible, is a safe and effective alternative that may result in long-term control with a minimum side effect profile.
